# Farmers’ indigenous knowledge in the management of coffee berry disease caused by *Colletotrichum kahawae* and its status assessment

**DOI:** 10.1186/s13002-026-00911-7

**Published:** 2026-06-01

**Authors:** Gerema Amente, Diriba Muleta, Fekadu Gadissa, Dagmawit Chombe, Kibrom B. Abreha, Cecilia Hammenhag, Kassahun Tesfaye, Mulatu Geleta

**Affiliations:** 1 Oromia Agricultural Research Institute, Adami Tulu Agricultural Research Center (ATARC), Adami Tullu-Batu, Ethiopia; 2 Biotechnology Research Center, Addis Ababa University, Addis Ababa, Ethiopia; 3 Department of Biology, Madda Walabu University, Bale-Robe, Ethiopia; 4 Bio and Emerging Technology Institute (BETin), Addis Ababa, Ethiopia; 5 Department of Plant Breeding, Swedish University of Agricultural Sciences (SLU), Alnarp, Sweden

**Keywords:** Arabica coffee (coffea arabica), Coffee berry disease, Indigenous knowledge systems, Disease incidence and severity, Participatory selection

## Abstract

**Background:**

Coffee berry disease (CBD), caused by *Colletotrichum kahawae*, is a major constraint to *Coffea arabica* production in Ethiopia, where coffee is crucial for livelihoods and national income. However, farmers’ indigenous knowledge and local disease management practices remain underexplored. This study aimed to document farmers’ knowledge of CBD detection and control and to assess disease incidence and severity across forest and semi-forest coffee systems along altitudinal gradients.

**Methods:**

A mixed-methods design was used, including structured household surveys of 90 experienced farmers across nine sites, focus group discussions (FGDs), key informant interviews (KIIs), and field assessments using berry count and visual scoring. Qualitative data were analysed thematically, while quantitative data on CBD incidence and severity were analysed using one-way ANOVA with Tukey’s HSD test (*p* < 0.05) in R (v4.5.3). Boxplots were used to compare variation across systems.

**Results:**

Nearly all farmers accurately identified early CBD symptoms through visual observation and relied heavily on indigenous knowledge for management. Common practices included pruning, sanitation (removal of infected parts), and selection of locally preferred coffee types. Farmers also reported selecting genotypes perceived as resistant based on berry traits, indicating informal farmer-led selection. However, these practices showed limited effectiveness in controlling disease spread. FGDs identified climate variability, dense canopy management, and weak institutional support as key drivers of CBD intensification. KIIs revealed gaps in access to resistant varieties, weak extension services, and limited integration of farmer selection criteria into formal breeding. Significant differences in CBD incidence and severity were observed across sites and production systems (*p* < 0.05), with forest systems showing higher severity and variability than semi-forest systems.

**Conclusions:**

Indigenous knowledge plays a vital role in early CBD detection and local adaptation strategies. However, current farmer practices alone are insufficient for effective control. Integrated approaches combining local knowledge, resistant varieties, improved agronomic practices, and strengthened extension services are essential for sustainable CBD management and conservation of Ethiopia’s Arabica coffee genetic resources.

## Introduction

The genus Coffea L. (Rubiaceae) comprises 103 species [13], among which *Coffea arabica* (arabica coffee) and *C. canephora* (robusta coffee) account for approximately 65% and 35% of the world’s coffee bean production, respectively [21]. Market preference is similarly dominated by arabica coffee, which represents the majority of the global demand. Arabica coffee, whose center of origin and genetic diversity is Ethiopia [20, 45], is more widely used than robusta coffee. This is mainly due to its unique qualities, such as a wide range of flavors, preferred aroma, and relatively low caffeine content, which are largely attributed to the high genetic diversity of its gene pool [8, 28]. It is the only tetraploid (2n = 4x = 44 chromosomes) species in the genus, resulting from a natural hybridization event between the diploid species *C. eugenioides* and *C. canephora* [18, 31].

*Coffea arabica* is cultivated in Ethiopia across a gradient of management intensities in forest, semi-forest, and garden systems, which dictate both the ecological structure and farmers’ intervention strategies [11]. Forest coffee (FC) represents a wild state with high biodiversity and minimal human impact, whereas the semi-forest coffee (SFC) system involves selective thinning of the canopy and understory to enhance light availability and yield [38]. Garden coffee (GC), the most intensive system, is typically located near homesteads and is characterized by high planting densities, regular pruning, and intercropping with food staples such as enset [29].

Despite its immense economic value to many countries worldwide, including Ethiopia, arabica coffee faces mounting challenges from abiotic and biotic stresses that continue to undermine productivity and threaten long-term sustainability [12]. Major coffee diseases include coffee berry disease (CBD), coffee wilt disease (CWD), and coffee leaf rust (CLR), caused by *Colletotrichum kahawae*, *Gibberella xylarioides*, and *Hemileia vastatrix*, respectively [2, 3, 14, 15, 30, 41].

CBD poses a significant threat to coffee production, particularly arabica coffee extensively grown in Ethiopia [4, 39]. The disease causes severe damage to coffee plants, resulting in substantial yield losses and economic hardship for smallholder farmers. As coffee management shifts from natural forests to domestic gardens, production scale and labor intensity increase, creating distinct microclimatic conditions that may significantly influence the prevalence and severity of diseases such as CBD [11, 38]. Previous studies have indicated that CBD can cause yield losses of up to 80%, leading to major economic losses for coffee producers, particularly smallholders [1, 6, 43]. A high prevalence of CBD in semi-forest coffee production systems has also been reported in Ethiopia [10]. Disease prevalence ranges from approximately 40% at lower altitudes to nearly 50% in coffee plantations located at mid-altitude forest sites. CBD intensity is influenced not only by altitude but also by microclimatic conditions such as high humidity and prolonged wetness, which frequently occur at lower altitudes and in valley bottoms and hollows [32]. Wariyo et al. [43] also reported higher CBD intensity at lower altitudes in southern Ethiopia, where elevated humidity aggravated disease spread. Previous studies further indicated that Ethiopian smallholder farmers employ diverse indigenous practices to reduce CBD severity [17, 37]. However, few studies have integrated these biophysical patterns with indigenous farmers’ knowledge across Ethiopia’s diverse forest, semi-forest, and garden coffee production systems.

Therefore, this study aimed to document local knowledge and practices related to CBD detection and management and to assess its incidence and severity across altitudinal gradients in Ethiopia.

## Research methodology

### Study sites and ethnographic context

**Fig. 1:**
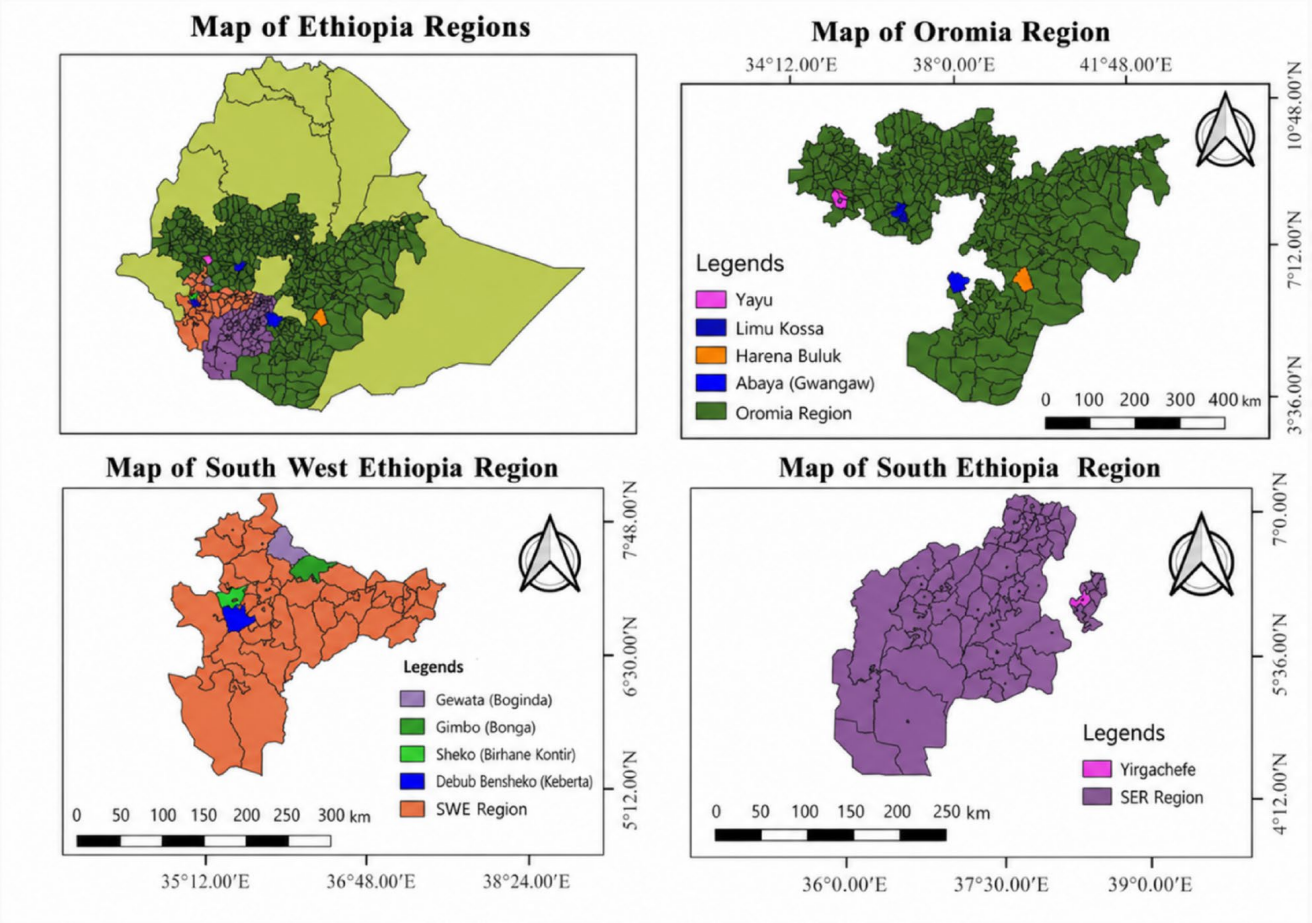
Location map of the study area in Ethiopia

This study was designed to represent Ethiopia’s diverse coffee-producing areas and systems. It covers nine well-established coffee-growing areas across three major administrative regions: Oromia, the Southwest Ethiopia Peoples’ Region (SWEPR), and the South Ethiopian Region (SER) (Fig. 1). In Oromia, Bale Zone (Harena Buluk), Jimma Zone (Limu Kosa), Ilubabor (Yayu)and Borena (Abaya-Gwangwa) sites were selected for their varied altitudes and microclimates. In the SWEPR, Sheko District (Birhane-Kontir), South Bench District (Keberta), Gimbo District (Bonga), and Gewata District (Boginda) sites were chosen to reflect differences in altitude and in rainfall. The Gedeo Zone (Yirgachefe) in southern Ethiopia, a region renowned for its high-quality coffee, was selected. The study areas were carefully selected to represent the main coffee production systems in Ethiopia: forest, semi-forest, and garden coffee. This classification was used to assess how differences in coffee genetic resources and management practices influence farmers’ knowledge and strategies for disease management.

Forest coffee grows naturally under forest conditions with minimal human intervention, mainly limited to harvesting and light understory clearing. Semi-forest coffee is managed more actively with practices such as shade thinning, removal of competing vegetation, pruning, and selection of productive plants. Garden coffee is cultivated near homesteads and farms under intensive management, including deliberate planting, closer spacing, regular weeding, pruning, mulching, shade regulation, and using organic inputs.

Interviews were conducted in Afan Oromo in the Oromia Region and Amharic in Southwest and South Ethiopia. The questionnaire was originally prepared in English and translated into the respective local languages during the interview process.

### Sampling strategy and data collection

A multi-tiered, mixed-methods approach was employed for primary data collection. A total of 90 experienced coffee farmers were recruited across the nine study sites (10 per site) using purposive sampling, based on recommendations from Development Agents (DAs) and community elders who identified farmers with the greatest breadth and depth of coffee production experience. This approach was not intended to produce a statistically representative sample of the farming population; rather, it was designed to capture the widest range of indigenous knowledge and practical experience relevant to CBD management. As a limitation, no information was collected to determine the proportion of sampled farmers relative to the total farm population at each site.

#### Household surveys

Structured and semi-structured questionnaires (Appendix 1) were administered to all 90 participants to obtain quantitative and qualitative data on: sociodemographic characteristics; farmers’ indigenous knowledge of CBD symptom recognition and management; perceptions of CBD trends and contributing factors; indigenous criteria for selection of CBD-resistant genotypes from forest coffee populations; and the socioeconomic significance of coffee production.

#### Focus group discussions (FGDs)

A total of nine FGDs were conducted, one per study site, each comprising 6–10 participants selected for diversity in sex, age, experience, and production system type. FGDs were guided by a structured protocol (Appendix 2) and lasted approximately 90–120 min. Each session was audio-recorded (with participant consent), transcribed verbatim, and translated into English. FGDs served primarily to validate, enrich, and triangulate household survey findings through collective deliberation, generating qualitative insights not readily captured by individual structured interviews.

#### Key informant interviews (KIIs)

In-depth KIIs were conducted with purposively selected informants at each study site (*n* = 27 total; 3 per site), including senior male and female farmers with long-standing coffee production experience, government DAs, cooperative leaders, local herbalists with ethno-botanical knowledge, and researchers from regional agricultural research stations. KII sessions followed a semi-structured guide (Appendix 3) and typically lasted 60–90 min. Responses were recorded, transcribed, and coded to extract key themes related to CBD knowledge, institutional gaps, and management innovation.

### Conceptualizing knowledge frameworks

This study distinguished two knowledge frameworks that shape farmers’ disease management decisions. Indigenous Knowledge (IK) encompasses traditional, experiential wisdom developed and transmitted across generations within local communities, relying on holistic phenotypic indicators including berry colouration, leaf texture, and plant vigor as diagnostic tools. Local Agricultural Knowledge (Institutional Knowledge) consists of standardized agronomic recommendations and extension packages provided by government DAs and regional agricultural offices, derived from formal scientific research and national coffee breeding programs. Both frameworks were documented and their complementarity assessed in the context of CBD management.

### Coffee production system descriptions

Forest coffee grows naturally under closed-canopy forest conditions with minimal human intervention beyond selective harvesting and limited understory clearing. Semi-forest coffee is managed more actively through shade thinning, pruning, selective retention of productive plants, and removal of competing vegetation to increase light penetration and yield. Garden coffee, cultivated adjacent to homesteads, represents the most intensive management system, characterized by deliberate planting, high planting density, systematic pruning, shade regulation, regular weeding and mulching, and organic input application.

### Methods employed for disease assessment

For Coffee berry disease (CBD) assessment, two complementary methods described by Van der Graaff [41, 42], berry count and visual estimation, were used.

#### Berry count method

The berry count method provides an accurate quantification of diseased berries on individual coffee trees. On each farm, five trees were randomly selected along a “W”-shaped path. Each tree was divided into three strata (top, middle, and bottom), and two middle branches with berries were selected from each stratum. The numbers of CBD-infected and healthy berries were counted. CBD-infected berries were differentiated from healthy berries based on visible symptoms, including dark, sunken lesions with sporulating fungal growth, whereas healthy berries presented no discoloration or surface damage. Care was taken to confirm that only berries with characteristic signs of Coffee berry disease were classified as diseased. The percentage of diseased berries was calculated using the following formula:

$$\begin{aligned}&\:Percentageofdiseasedberries\\ &\quad=\frac{Numberofdiseasedberries}{Totalnumberofsampledberries:}X100\end{aligned}$$

#### Visual assessment of CBD incidence

Disease incidence, defined as the proportion of individuals affected by a disease, is crucial for understanding the prevalence of disease in a population. Visual assessment is commonly used to determine the incidence of CBD. On each farm, ten coffee trees were randomly selected and visually inspected for CBD symptoms on the leaves and cherries. Each tree was scored as diseased or healthy, and the disease incidence was calculated as the percentage of diseased trees out of the total number of trees assessed.

$$\begin{aligned}&\:DiseaseIncidence\\ &\quad=\frac{Numberofinfectedtrees}{totalnumberoftreesaccessedonthefarm:}X100\end{aligned}$$

### Data analysis

Qualitative data from household surveys, FGDs, and KIIs were systematically organised and subjected to thematic analysis to identify recurring patterns, themes, and insights relevant to indigenous CBD knowledge and management practices. FGD and KII data were analysed separately and subsequently triangulated with survey data for convergent validation. The quantitative data, including berry counts and visual assessments of coffee berry disease severity and incidence, were analysed via R software (version 4.5.3). One-way analysis of variance (ANOVA) was used to assess differences in Coffee Berry Disease (CBD) incidence and severity among study sites, with site as the factor and incidence (%) and severity (%) as response variables. Mean comparisons were performed using Tukey’s HSD test at *p* < 0.05. The study sites were further grouped by production system to evaluate broader patterns, and box plots were used to visualize variation and trends.

## Results

### Sociodemographic characteristics of the respondents

The age of the surveyed household heads ranged from 20 to 81 years, with a mean of 45years, indicating that most respondents were in their mid-40 s (Table 1). The family size ranged from 1 to 14 members, with a mean of 7. The household dependency ratio varied between 0 and 5, with a mean of 1, which means the proportion of dependents (children under 15 and adults over 64) to the working-age population (typically 16–64). Years of experience in agriculture, specifically in coffee production, were highly correlated, ranging from to 5–66 years. The mean number of years of experience in agriculture was 27, whereas the mean number of years of experience in coffee production was 24 (Table 1). These results indicate that the households in the study area have substantial agricultural experience, particularly regarding coffee cultivation.

**Table 1:** Sociodemographic characteristics of surveyed household heads

Demographics of the surveyed households	Min.	Max.	Mean	SD
Age of household head	20	81	45	14
Family size less than 15 years	1	8	3	2
Family size between 15 and 64 years	2	12	4	2
Family size older than 64 years	1	3	0.4	1
Household family size	1	14	7	3
Dependency ratio	0	5	1	1
Experience in agricultural production	5	66	27	13
Experience in Coffee production in years	4	63	24	14
Land allocated to coffee cultivation (hec.)	0.25	6	1.2	1.1

Min= Minimum; Max= Maximum, SD= Standard Deviation

### Major challenges to coffee production

The ranking results indicate a clear hierarchy of constraints affecting coffee production (Table 2). Diseases and pests overwhelmingly dominated as the primary concern, receiving the highest proportion of Rank 1 and Rank 2 responses, confirming their critical and immediate impact. Erratic rainfall consistently appeared within the top three ranks, with a strong concentration at Rank 3 and Rank 2 underscoring its significant climatic influence. In contrast, the lack of improved varieties and poor farm management showed a more even distribution across ranks, suggesting moderate but widespread importance with variability among farmers. Rising temperatures were mainly positioned in the intermediate ranks (Ranks 3–5), reflecting an emerging but not yet dominant concern. Deforestation is largely concentrated in the mid-to-lower ranks (Ranks 5–8), indicating an indirect or longer-term effect on production systems. Price fluctuation was ranked among the lowest priorities (Ranks 7 and 8), highlighting its perception as an economic rather than an immediate agronomic constraint. Similarly, soil degradation was mostly assigned to lower-middle ranks, particularly Rank 6 suggesting that it is recognized as an underlying but less immediate challenge. Overall, the distribution pattern clearly distinguishes immediate biotic and climatic stresses from long-term environmental and economic constraints (Table 2).

**Table 2:** Ranking of coffee production challenges by respondent farmers (*n* = 90)

Challenges	Percentage of respondents (%)							
	Rank 1	Rank 2	Rank 3	Rank 4	Rank 5	Rank 6	Rank 7	Rank 8
Diseases and pests	63.6	19.5	2.6	3.9	2.6	7.8	0.0	0.0
Erratic rainfall	18.2	27.3	41.6	3.9	4.9	1.9	2.2	0
Lack of improved varieties	12.3	12.3	25.0	12.3	12.3	12.3	12.3	1.3
Poor farm management	10.4	22.1	10.4	27.3	7.8	6.5	5.2	10.3
Rising temperatures	2.6	15.6	23.4	27.3	14.3	1.3	2.6	12.9
Deforestation	2.6	3.9	5.2	6.5	29.9	15.6	16.9	19.4
Price fluctuation	1.3	1.3	2.6	9.1	5.2	14.3	37.7	28.5
Soil degradation	1.3	20.7	1.3	10.4	19.5	36.4	9.1	1.3

### Farmers’ indigenous knowledge on the detection and management of CBD

Among the 90 respondents surveyed, almost all farmers reported that they could identify coffee berry disease (CBD) symptoms, mainly at the early and infective stages (Table 3). The most commonly recognized symptoms included berry darkening and premature drying and dropping, whereas only a small proportion of respondents mentioned sunken berries. Symptom recognition was overwhelmingly based on visual observations. Most farmers indicated that they detect symptoms at an early stage, with only a few recognizing the disease after establishment or at peak infection (Table 4).

To manage disease spread, farmers employ a range of cultural practices that differ in their levels of intervention and specificity. Preventive measures, such as pruning, are commonly used to improve canopy structure, aeration, and light penetration, thereby reducing disease-conducive microclimates. In contrast, targeted phytosanitary practices include cutting and discarding infected plant parts, which involves the selective removal of visibly diseased tissues to limit pathogen spread. Similarly, removing infected berries combined with routine field management reflects an integrated approach aimed at reducing the inoculum while maintaining overall crop health.

More intensive interventions have also been reported. These included the complete removal of infected plants without immediate replacement, as well as removal followed by replanting with healthy seedlings, indicating efforts toward longer-term field rehabilitation. In some cases, farmers practiced the total removal and burning of infected plants to eliminate sources of infection. Less frequently, farmers reported fungicide application and simple physical removal of infected plants, suggesting limited reliance on chemical control and variations in resource availability.

Regarding the sources of these management strategies, the responses were mixed. Most farmers attributed their practices to indigenous knowledge and accumulated farming experience, whereas others relied on advice from agricultural extension agents.

**Table 3:** Knowledge and recognition of CBD symptoms

Question	Answer	Number of respondents (*N* = 90)	Percentage (%)
Knowledge of CBD symptoms	Yes	89	99
	No	1	1

**Table 4:** Farmers’ knowledge of coffee berry disease (CBD) symptoms, detection, and management practices (*N* = 90)

Category	Item	Number of respondents	Percentage (%)
Specific symptoms recognized	Sunken berries	8	9
	Berry darkening	80	89
	Premature drying and dropping	72	80
Symptom identification method	Visual inspection	87	96
	Other	3	4
Timing of symptom detection	Early onset	81	90
	After disease establishment	7	8
	At peak infection	1	1
	Other	1	1
Management measures used	Pruning	8	8
	Fungicide application	1	1
	Total removal and burning	8	9
	Removal and replacement of infected plants	8	8
	Physical removal of infected plants	3	3
	Removing infected berries and tending operations	9	10
	Cutting and discarding infected parts	12	14
Source of management practices	Development agents/Experts	33	37
	Farming experience/Indigenous knowledge	57	63

### Farmers’ success in managing coffee berry disease (CBD)

Among the surveyed farmers, only a small proportion reported success in controlling the spread of coffee berry disease (CBD) using their current management practices, whereas the vast majority indicated limited or no success (Table 5).

Despite this, approximately one-quarter of the respondents reported active involvement in selecting CBD-resistant genotypes from forest coffee populations, while the majority were not involved. Among those engaged in selection, the duration of involvement varied considerably, ranging from short-term experience (2–10 years) to long-term engagement (up to 30 years), with a notable proportion reporting more than two decades of experience.

Most farmers involved in genotype selection rated their efforts positively, with 44% describing their performance as *very good* and 39% as *good*, whereas a smaller proportion (rated it as *fair*. In terms of knowledge sources, more than half of the respondents relied on indigenous knowledge, whereas others followed the guidance of local agricultural experts.

The selection of resistant genotypes was predominantly based on berry color, which was identified by most farmers. Additional criteria, such as leaf and berry traits and the absence of visible symptoms, were reported by approximately one-quarter of respondents, while only a few used combined criteria.

The selection process was largely led by male elders, with minimal involvement of female elders and limited joint participation. This pattern was mainly attributed to differences in experience and exposure to coffee production practices, as reported by most respondents, while cultural factors were mentioned less frequently.

**Table 5:** Farmers’ success in managing coffee berry disease (CBD)

Question	Answer	Number of respondents (*N* = 90)	Percentage (%)
Have you been successful in controlling the spread of CBD through current measures?	No	86	96%
	Yes	4	4%
Were you involved in selecting CBD-resistant genotypes from forest coffee?	No	67	74%
	Yes	23	26%
If yes, for how long (in years)?	2	1	5%
	4	1	5%
	5	2	11%
	6	2	11%
	7	1	5%
	10	2	10%
	20	5	26%
	23	1	5%
	25	2	11%
	30	2	11%
Self-rated success in selecting resistant genotypes	Very good	10	44%
	Good	9	39%
	Fair	4	17%
Source of knowledge used during selection	Indigenous knowledge	14	58%
	Local agricultural experts	10	42%
Indigenous knowledge-based criteria used during selection	Leaf and berry colour	6	26%
	Berry colour	20	87%
	Absence of symptoms on berry	6	26%
	Berry colour and absence of symptoms on berry	1	4%
Who is largely involved in the selection process	Male elders	20	83%
	Female elders	1	4%
	Both	3	12%
Main reasons for largely involved in the selection process	Experience and exposure to the work	21	88%
	Cultural beliefs (tradition)	3	13%

### Respondents’ perceptions of coffee berry disease trends

**Fig. 2:**
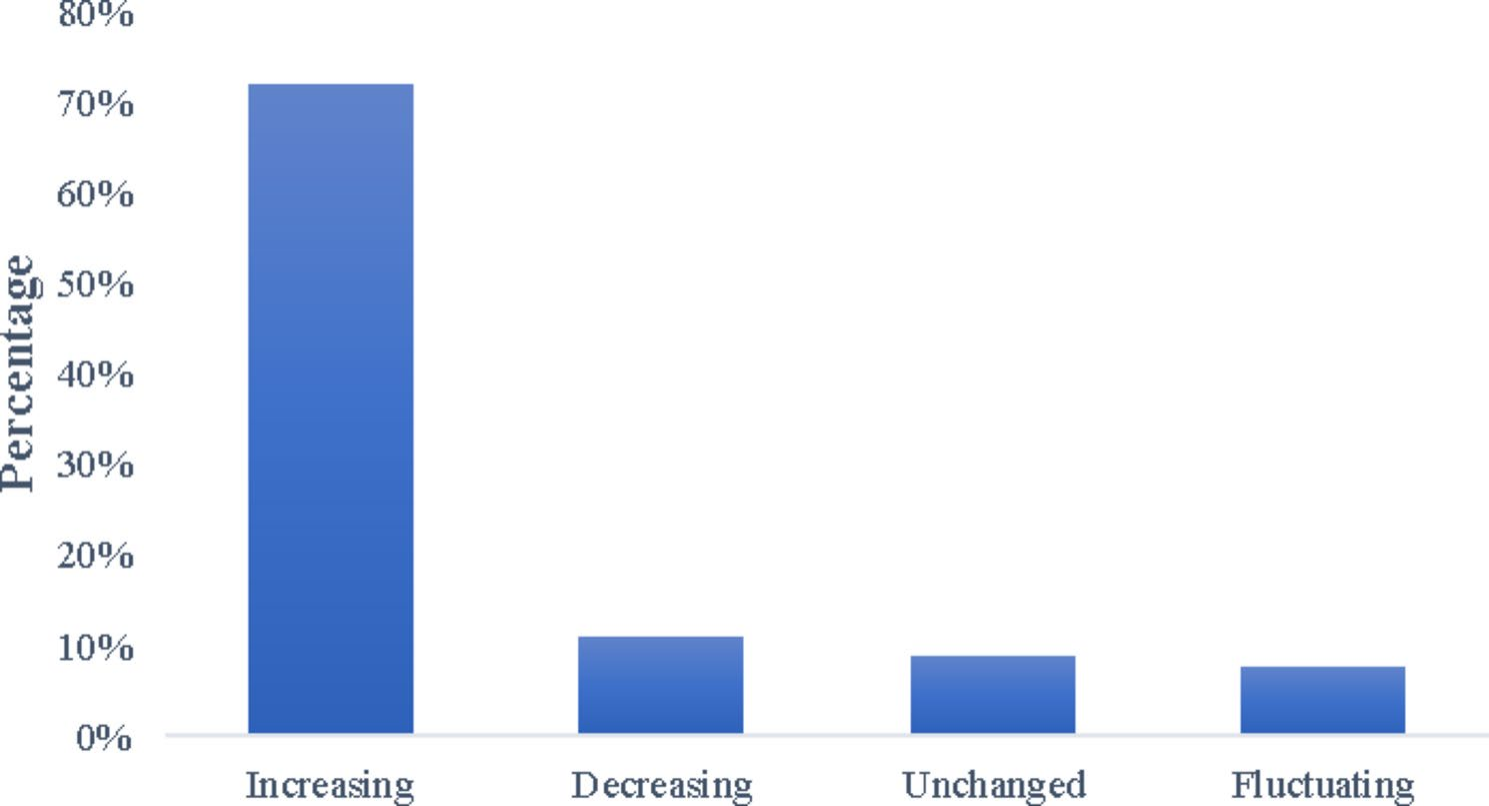
Respondents’ perceptions of trends in coffee berry disease (CBD)

The survey results indicate that CBD is widely perceived to be deteriorating in the study area. A significant majority of respondents (72%) reported an increasing prevalence of the disease, whereas 11% perceived a decreasing trend, 9% reported no change, and 8% noted fluctuations (Fig. 2).

**Fig. 3:**
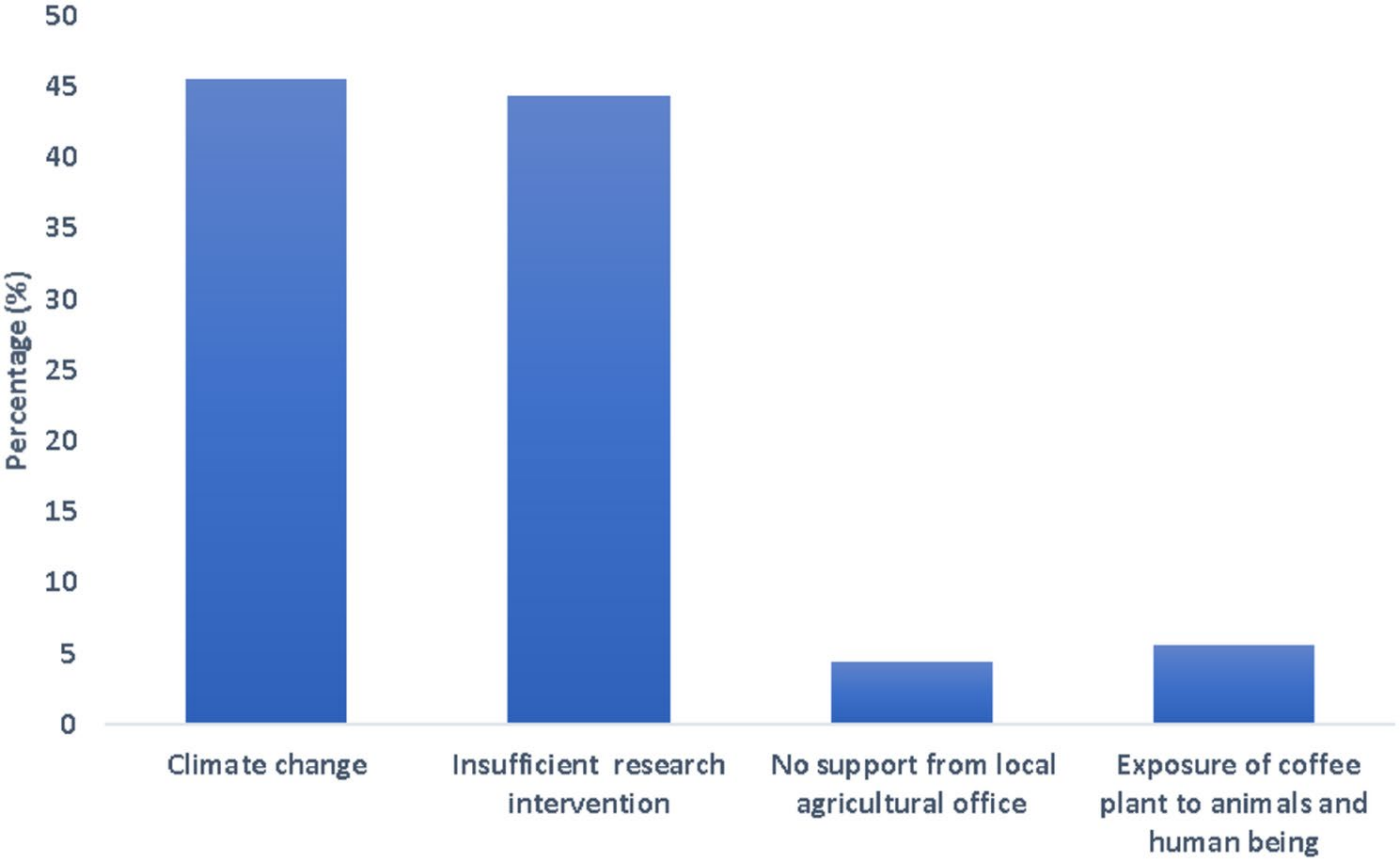
Key factors identified by respondents as contributing to the perceived increasing trend in coffee berry disease (CBD) prevalence

Regarding the major contributing factors to this trend, climate change was cited by 46% of the respondents, followed closely by a lack of research interventions (44%). Additional factors included the exposure of coffee plants to animal and human activities (6%) and insufficient support from local agricultural offices (4%) (Fig. 3).

**Fig. 4:**
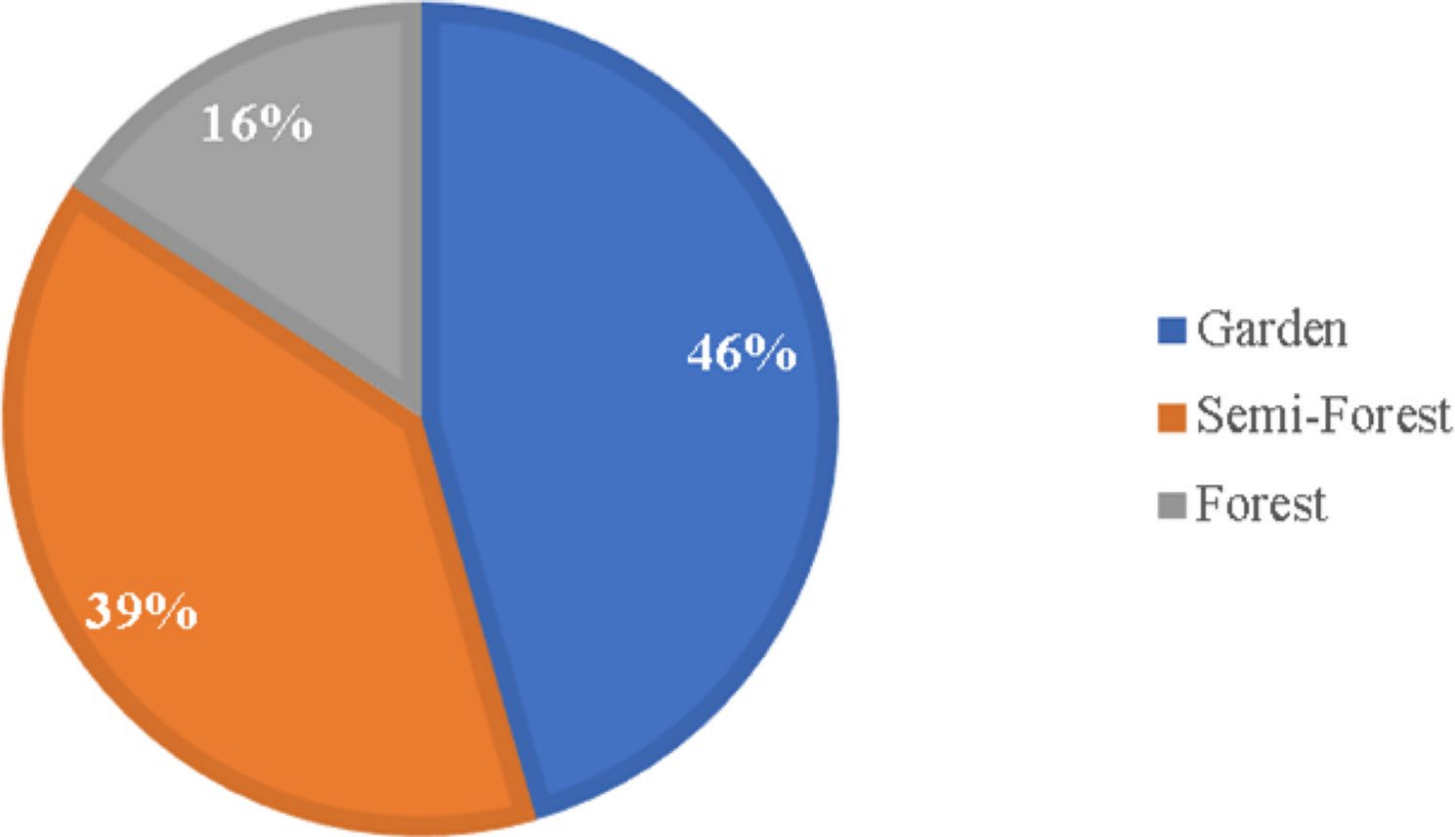
Coffee production system affected by coffee berry diseases in Ethiopia

In terms of coffee production systems affected by CBD, garden coffee was identified as the most impacted (46%), followed by semi-forest coffee (39%) and forest coffee (16%) (Fig. 4).

**Fig. 5:**
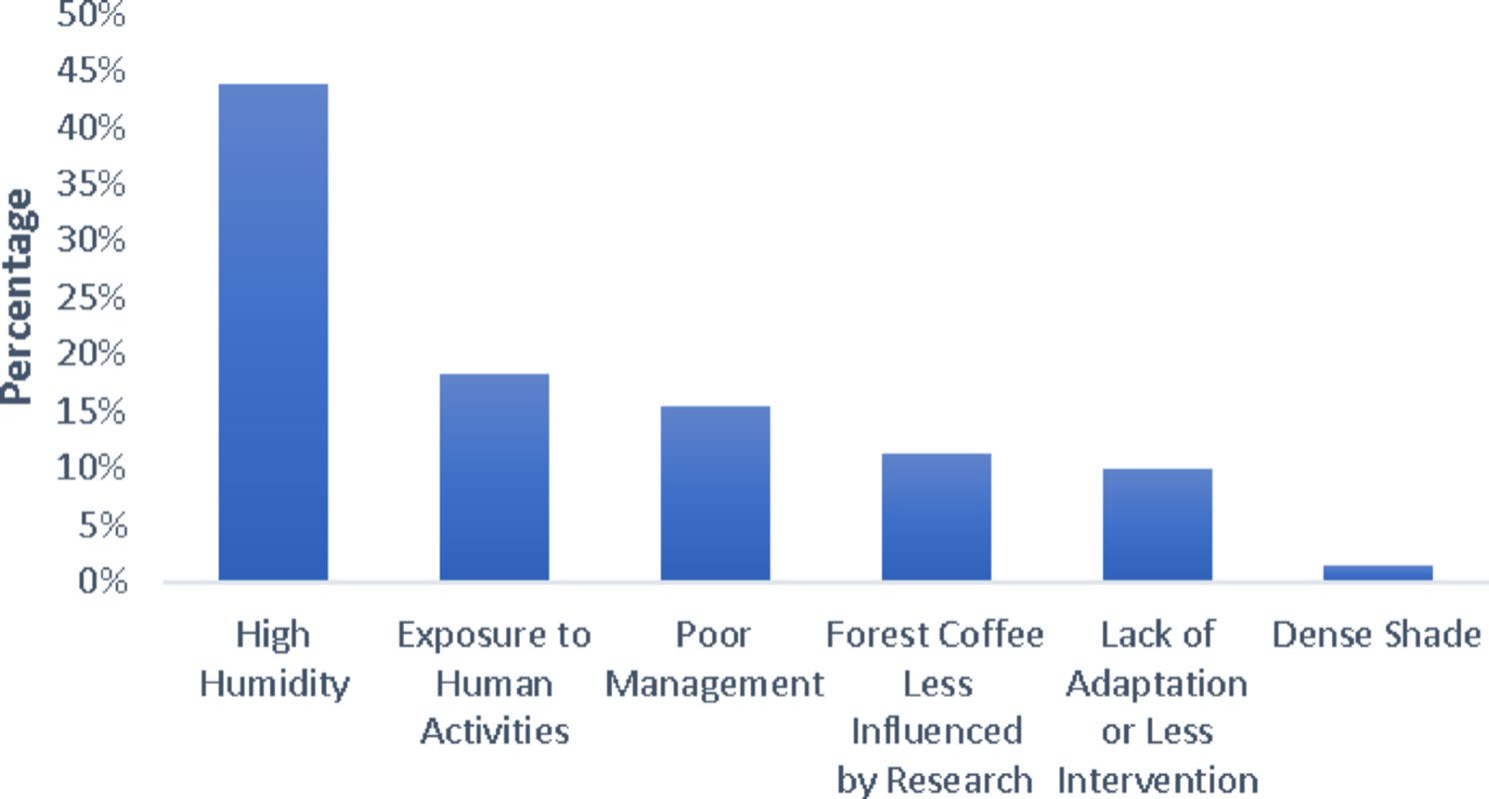
Major specific factors cited by respondents as contributors to the perceived increasing trend of CBD

The specific reasons for the perceived increasing trend of CBD included high humidity (44%), exposure to human activities (18%), poor management practices (16%), limited influence of research on forest coffee (11%), lack of intervention (10%), and dense shade (1%) (Fig. 5).

Analysis of farmer-reported disease management practices revealed that the total removal and burning of infected plants was the predominant coping strategy (45), followed by the practice of avoiding infected coffee berries (25). Other notable but less frequent methods included the selection of resistant varieties from local forest populations (15), general tending operations (10), and removal combined with replacement planting (5) (Fig. 5).

### Focus group discussion results

FGDs were conducted separately at each study site and yielded qualitative data that validated and substantially enriched household survey findings. The key thematic findings from FGDs, with representative verbatim statements (translated), are summarized in Table 6 below.

**Table 6:** Thematic summary of focus group discussion (FGD) findings across study sites

Theme	Key findings	Representative statements
**CBD symptom recognition**	Berry darkening and premature drop were universally recognized across all production systems	“We know the disease when the berries turn black and fall before harvest” (FGD, Bonga)
**Cultural management practices**	Pruning, sanitation harvesting, and shade management discussed as primary interventions	“We prune regularly after harvest to reduce humidity – it helps a little but does not stop the disease” (FGD, Yayu)
**Perceived disease trend**	Consensus that CBD is increasing; attributed primarily to changing rainfall patterns and inadequate institutional support	“The disease was manageable 20 years ago. Now it is everywhere. Rainfall is no longer predictable” (FGD, Limmu Kossa)
**Production system vulnerability**	Garden coffee perceived as most susceptible; forest coffee as relatively more resilient due to ecological balance	“In the forest, the disease does not spread as fast because the forest is balanced. In our gardens, once it starts, it takes over” (FGD, Harena Buluk)
**Gender roles in management**	Male elders dominate management decisions and genotype selection; women contribute to harvesting and monitoring	“The old men decide which trees to keep and which to remove. They have seen these trees for decades” (FGD, Boginda)
**Knowledge transmission**	Indigenous CBD knowledge transmitted orally from generation to generation; integration with extension advice is limited	“Our fathers taught us to identify diseased berries and remove them. Extension agents came once and told us to use chemicals, but where do we get them?” (FGD, Yirgacheffe)

One key cross-cutting finding from FGDs was the clear differentiation between forest and garden production systems. Farmers at all sites described forest systems as more ecologically buffered and less acutely disease-stressed than garden or semi-forest systems, due to natural biodiversity, shade continuity and lack of intensive human management. Participants in the FGD also said that traditional practices have become less effective over the last decade, which they attributed to worsening climate conditions and increasing population pressure on forest resources. FGDs also observed an increasing knowledge gap between male elders and younger generation farmers, which may lead to the loss of IK as younger farmers migrate to urban areas or use unconnected extension advice without traditional ecological basis.

### Key informant interview results

KIIs were conducted separately with 27 informants in the nine study sites and provided in-depth, expert level perspectives complementary to household survey and FGD data. The key findings from the KII informants, grouped by informant profile and knowledge domain, are presented in Table 7 below.

**Table 7:** Key informant interview (KII) findings by informant profile and knowledge domain

Informant profile	Domain of expertise	Key knowledge contributed
**Senior male elder (> 40 years coffee experience)**,** Boginda**	Long-term disease trend observation	Described progression of CBD from occasional disease to endemic status; identified altitude, moisture, and lack of resistant varieties as key drivers; reported reduction in forest coffee productivity over two decades
**Female elder farmer**,** Yayu**	Household-level disease monitoring	Provided detailed account of berry-based visual indicators used in early detection; described how harvesting practices evolved in response to CBD pressure; highlighted women’s role in day-to-day disease monitoring despite exclusion from formal selection activities
**Development Agent (DA)**,** Limmu Kossa**	Formal extension and technical knowledge	Described institutional recommendations for CBD control (variety distribution); acknowledged critical gap between official recommendations and farmer adoption due to limited supply chains and financial constraints
**Local herbalist/traditional practitioner**,** Harena Buluk**	Ethno-botanical knowledge integration	Shared use of local botanical preparations (plant extracts, ash applications) reported as supplemental practices to reduce disease incidence; noted diminishing use as younger generations adopt formal recommendations
**Coffee cooperative leader**,** Yirgacheffe**	Market and collective action knowledge	Discussed how CBD-affected produce receives lower market premiums; described collective efforts to establish disease-free seed plots; advocated for farmer-led certification of disease-tolerant local varieties
**Researcher/agronomist (government station)**,** Bonga**	Scientific assessment and resistance evaluation	Confirmed spatial variation in CBD pressure aligned with altitude and rainfall gradients; noted absence of dedicated resistant varieties for smallholder forest systems; recommended integration of farmer selection criteria into formal breeding programs

A particularly striking finding from KIIs was the agreement among researchers and DAs that the formal CBD-resistant variety distribution has been insufficient and spatially uneven, with many forest and semi-forest farmers reporting no access to improved planting material. In terms of KII participants, ethno-botanical experts stated that the usage of traditional botanical preparations for disease management declined due to social change and decreased inter-generational knowledge transfer, but not due to an inability to cure. Yirgacheffe cooperative leaders emphasized the economic incentive: CBD-free or low-CBD coffee fetches premium prices, thus providing a market-driven incentive for improved disease management, which extension programs have yet to tap into.

### Incidence and severity of coffee berry disease

**Fig. 6:**
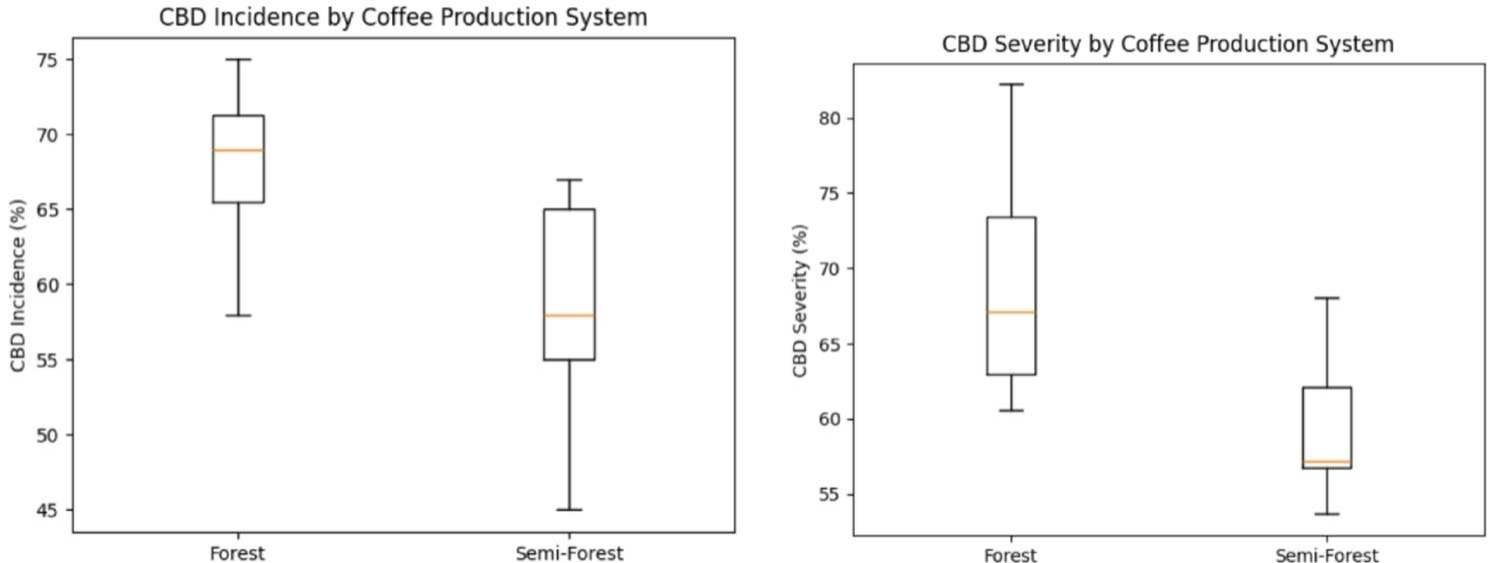
Boxplot showing variation in coffee berry disease (CBD) incidence (%) and severity (%) across coffee production systems

A significant difference in CBD incidence and severity was recorded across the study sites (*p* < 0.05), confirming a strong relationship between disease intensity and environmental factors (Table 8) (Fig. 6).

**Table 8:** Incidence and severity of CBD in major coffee-growing regions of Ethiopia

Study sites	Production type	Severity (%)	Incidence (%)	Altitude (m.a.s.l)	Annual rainfall (mm)	Annual temp (°C)
Boginda	Semi forest	57.19	65	1576	1094–1790	15–27 °C
Bonga	Forest	82.23	70	1737	1500–2500	15–25 °C
Keberta	Semi forest	53.67	45	1309	1400–1600	25–30 °C
Birhane Kontir	Forest	60.56	58	1383	1100–1300	17.5–20 °C
Yayu	Forest	70.54	75	1411	2000–2500	18–25 °C
Limmu Kossa	Semi forest	68.06	67	1598	1800–2000	18–22 °C
Harena Buluk	Forest	63.78	68	1676	1400–1900	4.6–26.8 °C.
Yirgachaffe	Semi forest	62.11	55	1568	1200–1400	16–21 °C
Gwangwa	Semi forest	56.79	58	1588	700–1200 m	19–23.5 °C

The present study revealed marked variation in Coffee berry disease (CBD) severity across Ethiopia’s forest and semi-forest coffee-growing regions, with values ranging from 54% to 82%. Bonga exhibited the highest severity (82%), followed by Yayu (71%), Limmu Kossa (68%), Harena Buluk (64%), Yirgacheffe (62%), Birhane Kontir (61%), Boginda (57%), Gwangwa (57%), and Keberta (54%). (Table 6).

Boxplot analysis revealed that the incidence and severity of CBD were generally higher in forest coffee systems than in semi-forest systems. The forest system also showed greater variability, indicating heterogeneous disease pressure across sites (Fig. 6).

## Discussion

### Sociodemographic characteristics of the respondents and their relevance to coffee management

The mean age of household heads observed in the present study suggests that the respondents are in a productive stage of life and are actively engaged in coffee management. This physical ability and long-term farming experience can be utilized to support broader coffee disease management practices. Similar findings were reported in the Wolaita Zone, where coffee farmers averaged in their mid 40 s, reflecting the accumulated knowledge and decision-making capacity that positively influenced production outcomes [40]. Similarly, Ngeywo et al. [35] in Kenya emphasized that middle-aged farmers are more productive because of their balance between energy and experience.

The relatively large mean family size highlights the centrality of coffee farming in sustaining the household livelihood. Tegegn et al. [40] reported that coffee production in southern Ethiopia is closely tied to household asset building and livelihood security, particularly in larger families that rely on coffee as their primary income source. Research in the Yayu Coffee Forest Biosphere Reserve also confirmed that coffee-based livelihoods play a critical role in sustaining household welfare and coping with demographic pressure [22].

The mean dependency ratio of 1 indicates that each working-age member supports just over one dependent, reflecting Ethiopia’s broader demographic structure, which is characterized by high fertility rates. While such ratios may strain household resources in the short term, they also suggest a future labor supply for agriculture, particularly in labour-intensive sectors such as coffee farming. As younger dependents transition into the workforce, they represent a demographic dividend that, if supported by targeted training, land access, and youth-inclusive policies, can enhance the sustainability and productivity of smallholder coffee systems.

### Major challenges to coffee production: conservation and breeding implications

The dominance of disease and pest infestation as the top-ranked challenge aligns with the literature on Ethiopian Arabica coffee. The prioritization of coffee–berry disease (CBD) reflects its devastating impact on yield and farmers’ livelihoods, as documented by Girma et al. [19] and Mulugeta et al. [34]. This concern highlights the urgency of developing resistant varieties and promoting integrated disease management strategies to control CBD.

Erratic rainfall was also reported as a major challenge (ranked second), indicating the sensitivity of coffee to climate variability. One of its consequences is that, owing to shifts in precipitation patterns, flowering and fruit development are disrupted, thereby significantly impacting local livelihoods and national earnings. Similar observations have been reported by Davis et al. [13] and Läderach et al. [25], who reported that erratic rainfall and shifting precipitation patterns severely disrupt coffee flowering and fruit development, thereby reducing yields and threatening both local livelihoods and national coffee revenues. Therefore, adopting climate-resilient agronomic practices, developing climate-resilient varieties, and improving local weather forecasting systems are essential for sustainable coffee production.

Although price fluctuation is a significant economic issue, its lower ranking than biophysical threats suggests that farmers prioritize immediate agronomic risks over market dynamics. This pattern is consistent with Wiersum et al. [44], who reported that smallholder farmers often focus on critical survival factors before optimizing their economic activities.

Moderate concerns regarding soil degradation, deforestation, and a lack of improved varieties reflect the recognition of long-term sustainability challenges. Although not perceived as immediate threats, these issues are crucial for strategic planning and policy intervention. The concern linked to poor management of coffee trees and rising temperatures further suggests that farmers are aware of both the technical and environmental constraints affecting productivity.

### Indigenous knowledge of CBD detection and management: implications for coffee breeding and conservation

This study demonstrates the strong indigenous knowledge Ethiopian coffee farmers possess regarding the identification and management of coffee berry disease (CBD). Nearly all respondents (99%) were able to recognize CBD symptoms at an early stage. The most commonly observed symptoms, berry darkening and premature fruit drop, are consistent with findings reported from southwestern Ethiopia [7]. The widespread use of visual inspection (96%) also reflects farmers’ long-term experience with coffee production and disease observation, highlighting the importance of farmer-based disease monitoring systems.

The high level of CBD recognition among farmers appears to result from years of practical experience and knowledge passed down across generations. Similar observations were reported by Bersisa et al. [9], where most farmers identified CBD through visual assessment. In many areas, this indigenous knowledge has become especially important because of the limited use of chemical control measures, helping farmers reduce disease spread under resource-constrained conditions [5].

Farmers mainly relied on cultural management practices such as cutting and discarding infected plant parts (14%), removing diseased berries (10%), burning infected materials (9%), pruning (8%), and replacing severely affected plants (8%). Similar dependence on non-chemical management strategies was also reported by Liebig et al. [26]. Fungicide application was rarely practiced (1%), which agrees with reports from southwestern Ethiopia [7]. The low use of resistant varieties and fungicides may largely be associated with poor access to improved planting materials and limited extension support [23].

The findings suggest that strengthening participatory breeding programs, farmer-led seed systems, and extension services could improve CBD management while conserving Ethiopia’s Arabica coffee diversity. Participatory breeding approaches have already shown success by combining farmer preferences with scientific selection methods to promote resistant varieties [36, 46]. In addition, farmer-managed seed systems have helped maintain locally adapted and disease-tolerant coffee landraces [23].

The study also indicates that farmers depend on both indigenous knowledge and agricultural experts, creating a hybrid learning system within the community. Farmers’ understanding of symptom development and their adaptive responses, such as early detection and selective pruning, demonstrate the value of locally adapted management practices for CBD control.

Cultural practices such as pruning, field sanitation, and removal of infected berries remain central to CBD management, particularly in forest and semi-forest coffee production systems where external interventions are limited.

Overall, these findings emphasize the need for location-specific and context-based CBD management strategies. According to Ayalew et al. [7], CBD incidence and coffee yield are strongly influenced by climate, canopy cover, and cultivar resistance. In such conditions, farmers’ reliance on visual assessment and cultural practices provides an important foundation for sustainable and integrated disease management.

### Farmers’ practices in managing CBDs and their implications for sustainability

The reported low success rate (4%) in controlling CBD highlights the continuing challenge posed by *Colletotrichum kahawae*. Climate variability, particularly changes in rainfall patterns and increased humidity, has further intensified disease spread [25]. Previous studies have shown that CBD severity is often greater in garden coffee systems because of lower ecological diversity, whereas forest-based systems tend to provide better natural resilience [24]. The present findings suggest that intensively managed coffee farms may be more ecologically vulnerable than previously assumed, emphasizing the environmental risks affecting these production systems.

Despite the limited effectiveness of conventional management practices, farmer-led selection of resistant coffee genotypes remains an important adaptive strategy for reducing CBD impacts. Farmers mainly rely on indigenous knowledge and visual indicators, such as berry appearance and the absence of symptoms, to identify tolerant plants. These practices are closely linked to Ethiopia’s rich Arabica coffee genetic diversity and the limited dependence on synthetic fungicides, making traditional selection approaches valuable for developing durable resistance. Unlike formal breeding programs, which often require long periods of evaluation, farmer-based seed selection provides a continuous and locally adapted process based on field performance and practical experience.

Community-based seed systems also play an important role in maintaining genetic diversity and improving disease tolerance. However, the dominance of elderly men (83%) in genotype selection reflects broader gender inequalities in Ethiopian agriculture. Although women contribute substantially to coffee production activities, their limited participation in selection and decision-making may restrict inclusive innovation. Strengthening participatory training programs and inclusive extension services could therefore enhance community-based CBD management and improve the long-term sustainability of coffee production systems.

### Current status and impacts of coffee berry disease on Ethiopian coffee farming systems

The survey confirmed that 72% of respondents reported an increasing trend in Coffee Berry Disease (CBD) prevalence, consistent with wider observations from East Africa. Wariyo et al. [37] reported a strong rise in CBD intensity across southern Ethiopia, with incidence ranging from 24.7 to 74.8% and severity from 15.7 to 53.4%. Similarly, Bersisa et al. [9] found high CBD levels in western Oromia, including 73% prevalence in Sasiga. Adem [10] reported wide variation in West Hararge (8.0–84.0%, mean 49.7%). In Uganda, Matovu et al. [27] documented outbreaks in high-altitude districts, while Mulinge [33] showed that pathogen abundance increases with altitude in Kenya. Alemu [5] and [45] further emphasized that CBD is most severe in high-altitude areas with favorable microclimates.

The identification of climate change and weak research intervention as major drivers of CBD increase aligns with Ayalew et al. [7], who reported that rising minimum temperatures during fruit development significantly increase disease incidence. In addition, poor pruning and dense canopy cover intensify disease pressure, creating challenges in balancing yield and disease control. Adaptive options such as selecting shade trees with staggered leaf fall have been suggested to reduce risk while maintaining productivity [38].

Institutional constraints also contribute to the problem. Farmers reported limited support from extension services and increased exposure of coffee plants to animals and human activities. These weaknesses reflect gaps in extension delivery, landscape coordination, and farmer training. Matovu et al. [27] also noted that poor agronomic practices such as lack of pruning, stumping, and fertilization increase vulnerability to CBD and coffee leaf rust, especially in older plantations.

CBD severity also varies across production systems. Garden coffee was reported as the most affected, followed by semi-forest and forest systems. Similar patterns have been observed in Uganda, where disease levels are generally higher under less shaded conditions. Although some studies reported no significant statistical differences across shade levels, reduced splash dispersal under dense canopy suggests that shade still plays an important role in limiting pathogen spread [32]. The higher vulnerability of garden coffee is also linked to microclimatic instability and greater human disturbance [16].

Farmers identified high humidity, human activity, poor management practices, limited research attention to forest coffee, lack of adaptation measures, and dense shade as key factors driving CBD outbreaks. These align with ecological drivers reported in Uganda and Kenya, where rainfall patterns, leaf wetness duration, and environmental conditions strongly influence infection dynamics [33, 27].

Current farmer responses are largely reactive. The most common practices include uprooting and burning infected plants, removing diseased berries, selecting tolerant plants from forest populations, and replanting. While these actions provide short-term control, they are not sufficient for long-term sustainability. Alemu [5] and Matovu et al. [27] emphasize that host resistance remains the most cost-effective strategy, particularly for smallholder farmers. However, challenges such as limited access to improved varieties and pathogen adaptability continue to constrain effective long-term control, further reinforcing the importance of resistance-based breeding strategies.

### Incidence, severity and spatial patterns of coffee–berry disease

The findings on CBD incidence confirm its widespread prevalence across Ethiopia’s major coffee-producing regions and highlight strong spatial variation in disease pressure. The observed incidence levels are higher than earlier reports by Alemu [5], who documented 40% in Bonga, 26.3% in Yayu, 18.6% in Harena, and 6% in Berhane-Kontir. Similarly, Adem and Amin [10] reported a wide range of 30% to 93.3% within a district, while Bersisa et al. [10] found an overall mean incidence of 49.9% in Arsi.

The spatial variation in CBD incidence reflects the interaction of ecological and agronomic factors. High-incidence areas such as Bonga, Harena Buluk, and Limmu Kossa are located at higher altitudes (> 1500 m.a.s.l.) with high rainfall (1800–2500 mm), creating favorable conditions for *Colletotrichum kahawae* infection and spread. These areas may also be affected by susceptible coffee genotypes and management limitations such as poor pruning and canopy regulation, which increase disease pressure.

In contrast, Keberta showed the lowest incidence, likely due to its lower altitude (1309 m.a.s.l.), warmer temperatures (25–30 °C), and relatively drier conditions (1400–1600 mm rainfall), which may limit pathogen development or reflect better adaptation of local management practices.

Moderate incidence levels observed in Birhane Kontir, Gwangwa, and Yirgacheffe suggest transitional environments where disease pressure exists but is less severe, likely influenced by intermediate altitude, rainfall, and canopy structure. These patterns highlight the importance of site-specific monitoring and targeted management strategies that integrate farmer knowledge with ecological factors such as shade management, pruning, and varietal selection.

CBD severity results also align with earlier studies [5, 10, 9], confirming its persistent and spatially variable nature across Ethiopia’s coffee landscapes. However, the present findings indicate relatively higher severity levels than previously reported, suggesting an increasing disease burden over time.

High-severity areas such as Bonga and Yayu are characterized by dense canopy cover, high rainfall, and cooler temperatures, conditions that favor pathogen sporulation and spread. Similar observations were made by Alemu [5] and Desalegn et al. [36], while Tesfaye et al. [37] reported that canopy thinning can significantly reduce disease severity in comparable environments.

Lower severity in Keberta and Gwangwa may be associated with warmer and less humid conditions, which are less favorable for pathogen development, although CBD remains present even in these areas.

The combined evidence from field data, FGDs, and KIIs indicates a consistent trend of increasing CBD pressure across Ethiopia’s coffee systems, particularly in high-altitude and high-rainfall zones and in garden coffee systems with limited ecological buffering. Farmers’ perceptions (72% reporting increasing prevalence), alongside identified drivers such as climate change, poor canopy management, and weak institutional support, reinforce this trend.

## Conclusions and recommendations

This study confirms that coffee berry disease (CBD) is a growing threat to Ethiopia’s Arabica coffee sector, particularly in forest and semi-forest systems across diverse altitudes. The disease continues to overwhelm current management practices, placing increasing pressure on vulnerable production zones, especially high-altitude, high-rainfall areas, where microclimatic conditions strongly favor pathogen development. Most farmers have a strong capacity to detect CBD symptoms and apply local practices. However, conventional culture methods alone have proven largely ineffective in controlling the disease and are especially limited in garden coffee systems, which show increased susceptibility due to microclimatic instability and human disturbance.

Equally important is the need to strengthen farmers’ training not only in basic cultural practices, such as pruning and shade regulation, but also in site-specific management strategies that integrate indigenous knowledge, adaptive shade-tree selection, and improved canopy management. These approaches are essential for reducing humidity and splash dispersal, thereby creating microclimates that are less conducive to disease development.

Finally, sustained investment in agricultural research and extension services is essential. This includes expanding access to resistant cultivars, conducting location-specific disease surveillance, and addressing institutional gaps that limit farmers’ ability to adopt improved practices. Without consistent support, the gap between local knowledge and formal intervention will persist, undermining efforts to protect Ethiopia’s unique coffee genetic resources and the livelihoods they sustain in the process.

## Data Availability

All data generated or analyzed during this study are included in this article file. The dataset consists of farmers’ responses summarized as percentages, means, and frequency distributions. Raw interview data are not publicly available due to confidentiality agreements with participants, but aggregated results are fully presented in the manuscript.
